# Responsive and resilient healthcare? ‘Moments of Resilience’ in post-hospitalisation services for COVID-19

**DOI:** 10.1186/s12913-023-09645-8

**Published:** 2023-07-03

**Authors:** Charlotte Overton, Tristan Emerson, Rachael A Evans, Natalie Armstrong

**Affiliations:** 1 Department of Population Health Sciences, University of Leicester, Leicester, UK; 2 Leicester NIHR Biomedical Research Centre - Respiratory, University Hospitals of Leicester NHS Trust, Leicester, UK; 3 Department of Respiratory Sciences, University of Leicester, Leicester, UK

**Keywords:** Resilience framework, Healthcare, Long covid, Qualitative

## Abstract

**Background:**

COVID-19 caused disruption to healthcare services globally, resulting in high numbers of hospital admissions and with those discharged often requiring ongoing support. Within the UK, post-discharge services typically developed organically and were shaped over time by local need, funding, and government guidance. Drawing on the Moments of Resilience framework, we explore the development of follow-up services for hospitalised patients by considering the links between resilience at different system levels over time. This study contributes to the resilient healthcare literature by providing empirical evidence of how diverse stakeholders developed and adapted services for patients following hospitalisation with COVID-19 and how action taken at one system level influenced another.

**Methods:**

Qualitative research comprising comparative case studies based on interviews. Across three purposively selected case studies (two in England, one in Wales) a total of 33 semi-structured interviews were conducted with clinical staff, managers and commissioners who had been involved in developing and/or implementing post-hospitalisation follow-up services. The interviews were audio-recorded and professionally transcribed. Analysis was conducted with the aid of NVivo 12.

**Results:**

Case studies demonstrated three distinct examples of how healthcare organisations developed and adapted their post-discharge care provision for patients, post-hospitalisation with COVID-19. Initially, the moral distress of witnessing the impact of COVID-19 on patients who were being discharged coupled with local demand gave clinical staff the impetus to take action. Clinical staff and managers worked closely to plan and deliver organisations’ responses. Funding availability and other contextual factors influenced situated and immediate responses and structural adaptations to the post-hospitalisation services. As the pandemic evolved, NHS England and the Welsh government provided funding and guidance for systemic adaptations to post-COVID assessment clinics. Over time, adaptations made at the situated, structural, and systemic levels influenced the resilience and sustainability of services.

**Conclusions:**

This paper addresses understudied, yet inherently important, aspects of resilience in healthcare by exploring when and where resilience occurs across the healthcare system and how action taken at one system level influenced another. Comparison across the case studies showed that organisations responded in similar and different ways and on varying timescales to a disruption and national level strategies.

## Introduction

COVID-19 has challenged healthcare systems globally, with services having to identify, respond and adapt to unprecedented and dynamic changes in both healthcare needs and in how healthcare is organised and delivered. Key to effective response and successful adaption to these significant challenges is likely to be an organisation’s level of resilience.

The concept of resilience as the measure of disruption a system can absorb has been applied to a range of complex adaptive systems in multiple sectors (e.g. health, aviation, finance) and research disciplines (e.g. psychology, engineering, ecology) [1, 2]. Resilience is inherently a systems-orientated concept, from individual cognition to entire societies, and can be used to examine the complex interrelations and interconnections between different levels and scales of these systems [3]. It has been theorised in different ways, leading to multiple definitions that are contested and debated [4, 5], but it broadly refers to the capacity of a system to handle disruptions, failures and surprises in ways that avoid total system collapse, and may lead to adaptation and improvement [6].

In healthcare research, the concept of resilience focuses attention on the nature of the healthcare system and the adaptive work that is done to deliver safe care. Healthcare is a complex sociotechnical system with multiple and diverse interrelations and interconnections; consequently, when and where resilience occurs is inherently variable [6]. This conceptualisation of resilience depends on the interactions between stakeholders at different system levels, from healthcare professionals and patients at the sharp end to higher-order policy makers and regulators [2]. In their recent qualitative analysis on the impact of the COVID-19 pandemic on the concept of resilience, Paschoalotto et al. [7] argue that it is important to consider the context in which the shock happens. Resilience, therefore, is considered to be a time-specific, situated and contextualised matter [8, 9]. However, policymakers’ responses to shortcomings during times of crisis vary [7] and health systems’ actual capacity to anticipate and cope with uncertainties is dependent on access to flexible, adaptable resources [10], thereby highlighting the importance of understanding the interrelations and interconnections between different system levels on the emergence of resilience [3].

The need for a multi-level perspective in resilience research has been identified, but research has not thus far been able to adequately explain the links between resilience at different system levels or empirically investigate how action taken at one level may influence another [11].

## ‘Moments of Resilience’ in the development of post-hospitalisation services for COVID-19 patients

A pandemic introduces huge challenges and opportunities for both resilience and innovation in healthcare. Adaptations to ensure resilience in healthcare can take many forms, and the timeframe can be from seconds and minutes to long-term reorganisations that unfold over years and decades [6]. In this paper, we draw on Macrae’s Moments of Resilience framework, developed with the aim of building our understanding of resilience in complex sociotechnical systems. The framework considers resilience across different scales of organisational activity, in terms of scale and reach that unfold around a disruption. It can be used to examine the complex interrelations and interconnections between different levels and scales of complex sociotechnical systems.

Within the framework, [6] Macrae describes resilience in terms of time, space, organisations, and levels, providing a valuable structure for understanding resilience in time-sensitive situations, like a pandemic. The framework “characterises organisational activities as unfolding within three broad “moments” of resilience: situated, structural and systemic”(6:16–17). Situated resilience emerges at or close to the operational frontline and refers to how unexpected events are managed. It involves mobilising and combining existing sociotechnical resources (such as knowledge, skills, IT systems, data, clinic space) to detect, adjust to, and recover from disruptive events that occur in relatively small scales of time and space and can unfold over seconds to weeks. Structural resilience emerges in the monitoring of operational activities and is the process of redesigning and restructuring sociotechnical resources to adapt to or accommodate disruptive events to better support work. This can unfold over weeks to years. Systemic resilience emerges in the oversight of system structure and focusses on activities that involve reconfiguring or entirely reformulating how sociotechnical resources are designed, produced and organised. This can unfold over months to decades [6].

In this paper, we use the Moments of Resilience framework to inform a comparative analysis of three healthcare organisations’ development, implementation, and adaptation of post-hospitalisation services for patients following a hospital admission with COVID-19. Understanding such work as representing resilience through the ability to recover or adapt, we explore how these new services developed in response to emerging need and the factors influencing their design and implementation.

## Setting

Our study involved healthcare organisations from England and Wales. Since the start of the pandemic in March 2020, high numbers of patients have been hospitalised in these countries with COVID-19 [12, 13]. Survivors of hospitalisation with COVID-19 experienced unpredictable outcomes, with many reporting prolonged symptoms negatively impacting recovery [14]. Over the last two years, Long Covid has emerged as a significant healthcare issue. As of September 2022, an estimated two million people living in private households in the UK were experiencing self-reported Long Covid symptoms [15]. Reported symptoms are extensive, multidimensional, episodic and unpredictable in nature [16]. At least 65 million individuals around the world have Long Covid, based on a conservative estimated incidence of 10% of infected people and more than 651 million documented COVID-19 cases worldwide, the number is likely much higher due to many undocumented cases [17],

Multiple biomedical findings have been documented, with many patients experiencing dozens of symptoms across multiple organ systems [14]. As a complex multi-system condition with more than 200 symptoms, multiple adverse outcomes, with common new-onset conditions, Long Covid’s impact can be wide ranging – affecting a person’s physical, mental and psycho-social wellbeing [17].

The first specific guidance for management of patients with a radiological diagnosis of COVID-19 pneumonia was published by the British Thoracic Society in May 2020 [18]. The guidance was for patients who had been hospitalised or directly discharged from the emergency department or medical assessment unit [18]. In November 2020, NHS England published national guidance for post-COVID-19 syndrome assessment clinics (also referred to as Long Covid clinics), and in December 2020, the National Institute for Health and Care Excellence issued guidance on best practice for recognising, investigating and rehabilitating patients with Long Covid, irrespective of whether they had been hospitalised with COVID-19 or not [19].

Following its guidance for post-COVID-19 assessment clinics, in December 2020 NHS England announced funding for 69 such clinics [20]. For the 2022/23 financial year, £90 million has been committed for Long Covid services [21]. In England, the funding was allocated via Clinical Commissioning Groups (CCG) and guidelines for configuration of services were interpreted at a local level. As a result of local interpretation in the English context, clinics are led from both secondary care and community care settings [20].

The situation varied in Wales, with the Welsh government initially publishing a framework for rehabilitation services in May 2020 [22]. Funding for Long Covid services was allocated by the government in June 2021, via the Adferiad (Recovery) programme [23]. In Wales, the funding was allocated to Local Health Boards to provide a model of community-led rehabilitation services.

What is important to note is that, prior to government guidance and funding, there were no co-ordinated NHS commissioned follow-up services nor a clear model for how these should be developed and implemented [24]. This led to varied follow-up for patients following hospitalisation with COVID-19, with hospital and community teams using their own judgements (rather than research and guidelines) to make decisions about how to follow up patients, and doing so within existing resources [24, 25].

## Methods

Our research was part of a mixed-methods study with a wider aim to evaluate clinical and cost-effectiveness of healthcare pathways for adults discharged from hospital after COVID-19. In the current research, we aimed to identify how patterns of care post-hospitalisation developed and evolved through the pandemic from the first wave (March-July 2020) through the second wave (October 2020-March 2021). The mixed-methods study was affiliated with the Post-hospitalisation COVID-19 (PHOSP-COVID) study platform [26] and PHOSP-COVID sites were used for the current research. CO, TE and NA are affiliated with the wider PHOSP-COVID study; RAE is lead co-investigator. Our work package was qualitative in design, comprising comparative case studies [27] based on interviews. We sought to explore the experiences of healthcare professionals and managers of developing and implementing post-hospitalisation follow-up services, including barriers and facilitators and lessons learned. We also explored patients’ experiences of these services, but these are not reported in this paper.

We selected three healthcare organisations as case studies (see Table 1), using a typology of follow-up services developed earlier in the project and published elsewhere [28]. The typology focused both on the extent and range of follow-up services offered and whether these were available to all patients or only specific sub-groups (e.g. those who had been in intensive care) – a simple 2 × 2 matrix giving four categories. We originally planned to select one case study from each category, but due to time and capacity limitations were only able to complete three. We did not complete a case study of a limited service offered to only few patients. Case study selection within categories was purposive to include diversity in location and organisation type and size. For example, to ensure geographical diversity we purposively chose different regions in England and a site in Wales. Selecting for diversity in case studies allows new insights to be generated through exploration of the reasons for and consequences of inter-case differences [29].

**Table 1:** Case study organisations

Case Study 1 (CS1)	Teaching hospital in the Midlands; available to all post-hospitalised patients, holistic and multi-system approach, comprehensive rehabilitation; access to multiple mental health services
Case Study 2 (CS2)	Teaching hospital in London; available to all post-hospitalised patients, single organ focus, no rehabilitation; access to multiple mental health services
Case Study 3 (CS3)	District general hospital in a Health Board covering a large geographical area in Wales; available to pre-specified sub-group of post-hospitalised patients, single organ focus, access to rehabilitation in existing community services; no access to mental health services

We obtained ethical approval via an amendment to the PHOSP-COVID study (see Declarations).

The PHOSP-COVID Principal Investigator (PI) in each site was contacted to introduce the project. If they were not the post-hospitalisation service lead, they introduced the researchers via email to whoever was. The leads were interviewed in depth about their organisation’s approach to setting up a post-hospitalisation service. Purposive sampling and snowballing techniques were used to recruit further staff participants at each site, guided by the specifics of each post-hospitalisation service. Researchers e-mailed information sheets and consent forms before arranging a telephone or video call interview. We ensured participant information was clear that staff could decline to participate, and this was reiterated by researchers.

An interview guide developed by the study team was used to steer the interviews and was reviewed prior to use by the PHOSP-COVID Patient and Public Involvement group representatives, who also attended a subsequent presentation of findings. The guide was also reviewed by a Long Covid clinic lead. Participants were asked what happened when patients were discharged from hospital, to describe any services that were offered, how these had been developed and implemented, and if there were any plans to change or adapt the services. All interviews were conducted by non-clinical members of the research team (CO and TE), who were experienced qualitative researchers. Interviews lasted from 20 min to one hour and were audio-recorded.

In total, we contacted 38 healthcare staff and managers and conducted 33 interviews with 31 healthcare staff and managers between October 2021 and June 2022 (see Table 2). We interviewed staff at CS1 first, followed by CS2, then CS3. To gain an understanding of how services may have evolved over time, we asked staff to talk about when services were first set up through to the time of interview. As we interviewed staff at CS1 first, we interviewed two key members of staff again at the end of the data collection period.

**Table 2:** Staff by case study site and role

Case Study	Role	Number
CS1	Doctor (respiratory, cardiology, neurology, psychiatry, diabetology, General Practitioner)	6
	Nurse (respiratory, rehabilitation)	3
	Allied Health Professional (AHP) (physiotherapy)	1
	Commissioner	1
CS2	Doctor (respiratory)	3
	Nurse (Headache Clinical Nurse Specialist, Parkinson’s Clinical Nurse Specialist)	2
	AHP (physiotherapy)	3
	Manager	1
CS3	Doctor (respiratory)	1
	AHP (physiotherapy, occupational therapy, dietician, clinical psychologist)	5
	Manager	4
	Educator	1

Interview recordings were transcribed and imported into NVivo 12 software for coding and analysis. Our analysis used constant comparative and thematic analytic techniques that were consistent with the iterative approach we took to data collection [30, 31]. CO and TE conducted initial open coding of several transcripts and discussed emergent findings with NA. From this, a thematic framework was developed and used to code subsequent transcripts. Each researcher coded the interviews of the other, as well as a sample of their own. During analysis, CO, TE and NA met weekly to reflect on data and discuss findings.

## Findings

The broader context of the pandemic had created a new situation in healthcare, with services in both secondary and primary care under unprecedented pressure and having to adapt to working in different ways. The ongoing impact of COVID-19 after hospitalisation emerged over time and caused disruption for the whole healthcare system. This ranged from the level of clinicians working at the operational frontline, to managers and commissioners who oversaw the development of new services, and to governmental level where decisions were made about funding and guidance for follow-up services. Our findings show that moments of resilience occurred as a multi-layered set of related processes enacted over different time periods and at different levels of activity. Across all sites, the service design was influenced by contextual and demographic factors. As such, we found similarities and differences in how patients were followed up after being discharged from hospital.

In this section, we illustrate the dynamic interactions of people and their immediate work environment and the adaptation, adjustment and intelligence required to respond to the impact of COVID-19 on patients discharged from hospital.

### Initial response to patients being discharged with on-going issues

At each case study site, respiratory consultants, specialist nurses and physiotherapists working at the operational frontline identified that COVID-19 patients were being discharged home with complex ongoing issues. In addition, evidence was emerging that patients were not recovering from acute COVID-19 as expected. From a level of individual cognition, staff spoke of moral distress related to witnessing the impact of COVID-19, and a sense that patients should not be suffering. This resulted in a reaction of motivation to take action.

*I said to my colleagues “Goodness, we’ve sent home patients with abnormal blood tests, abnormal x-rays, with no follow up and that’s not we would have done with a pneumonia usually. This is really awful”, so it’s fortunate that I’m with likeminded individuals and it was felt that we did need to follow up these patients in some way*. **CS2 Service Lead**

In healthcare, service delivery models are usually designed, delivered and adapted within a framework of research, policy, standards, and protocols, with any deviations being based on clinical expertise and prior knowledge [32]. Very little was known about Long Covid when services were first set up, so sociotechnical resources such as knowledge and skills were used to design the service and in the day-to-day delivery of the service at the operational frontline [24]. With the aim of taking a proactive approach, drawing on a variety of sources created moments of situated resilience at the micro-level, clinical leads approached colleagues with existing knowledge from the requisite specialisms that aligned with knowledge from clinical practice and research on the emerging sequelae of Long Covid. Requests were made to give advice on screening for symptoms that patients were presenting with outside of their expertise in respiratory medicine.

*I started having conversations with my consultant colleagues within respiratory medicine. We then reached out to the Infectious Diseases team, cardiology, renal, because it was clearly a multisystem disease. And we also reached out to colleagues at other hospitals and settled on some tests that we felt were necessary and set some questions that we thought were important to ask and to screen for*. **CS2 Service Lead**

Following the initial response, respiratory clinical staff recognised that existing resources would not be sufficient to address the scale of the disruption. Accordingly, staff at each site escalated the problem to managers at the meso level who were in a position to authorise the necessary structural adaptations. This included the reorganisation of the sociotechnical resources (staff, clinic space, IT, knowledge and skills) required to set up a new service at the micro level. Strategies differed between sites and were influenced by the availability of sociotechnical resources, contextual and demographic factors.

At CS1 and CS2 early in the pandemic, in 2020, there was a favourable response from managers at the meso level to the adverse event created by the ongoing impact of COVID-19 on a significant number of discharged patients, and follow-up services were set-up. It was unprecedented to set up a new service for a new condition in the broader context of a pandemic, and multiple actors were needed to accomplish this. Clinical staff and managers were motivated to be involved by a sense of goodwill, working together to support each other, and wanting to ‘do the right thing.’

*I soon realised that actually it was a really important thing that we were doing, it [the service] was going to make a massive difference to get it right and to implement it and to hit the floor running with it really because the evidence that was coming out of people that had been discharged without any kind of support in the community was developing complex issues and needed us, so that sort of made me feel like I could actually make a difference*. **CS1 Nurse**

*It was really great to see everybody just coming together and working hard and it didn’t matter what specialty you were from and what discipline of professional you were, so that was really, I mean it was truly and utterly team-working at its best*. **CS2 Service Lead**

CS3 saw fewer patients admitted in waves one and two than at CS1 and CS2. Early on in the pandemic, moments of situated resilience at the meso-level at CS3 differed from those seen at CS1 and CS2. While a CS3 respiratory consultant argued that a strategy needed to be in place to provide follow-up for discharged patients, managers within this case study (who would have been able to redesign and restructure the sociotechnical resources required to set up a service) did not believe there was sufficient demand to justify funding a specific post-hospitalisation follow-up service from existing budgets. So, at this time, a service specifically for the follow-up of discharged patients was not set up, and patients with on-going issues were referred to existing community rehabilitation services or remained under the care of secondary care clinicians. The respiratory consultant believed that not providing a specific service was inadequate and, in response, reacted by adapting existing resources under their control to provide follow-up for some patients in an out-patient clinic. Adapting and adjusting the sociotechnical resources available to him allowed moments of situated resilience to emerge, but the consultant was cognisant of the limitations of such an approach.

*For those patients who were pretty ill on our COVID ward we offered them a targeted respiratory clinic follow and these are people with severe lung disease who needed ITU or CPAP and they had a specific respiratory follow up only, but nothing for generic COVID and those without organ damage or normal x-rays on discharge had nothing*. **CS3 Doctor**

Managers at CS3 continued to monitor operational activity and by mid-2021, the number of referrals to community-based rehabilitation services for discharged hospitalised patients with on-going issues was creating disruption in those existing services. At this point, managers were mobilised to take action and designed a Long Covid service to accommodate the impact caused across the Health Board. Moments of structural resilience were represented through the active processes of designing a follow-up service in light of this disruptive event.

At each case site, the enactment of the structural adaptations required to enrol sociotechnical resources was initiated from situated practices that unfolded around the disruptive event of patients being discharged from hospital with on-going issues. Although at different times in the pandemic, at each site in response to contextual and demographic factors the application of sociotechnical resources created moments of situated resilience at the micro-level.

### The redesign and restructure of existing resources to set up follow-up services

The local disruption identified by respiratory consultants, specialist nurses and physiotherapists led to operational activity at the meso-level. This involved the redesign and restructuring of existing sociotechnical resources required at the situated level to set-up and deliver a new follow-up service. For example, across all case study sites, sociotechnical resources such as established and new IT systems were developed and adapted to monitor patients discharged from hospital.

*We knew that we needed to catch these patients because they were going to need long term management potentially, so fortunately we had the foresight of setting up databases to capture these people, put plans in place to try and monitor them and support them*. **CS1 Nurse**

The work that usually occurs to set up a service happened at a quicker pace, with adaptations being made to working practices. At CS2, the provision of funding from existing budgets to set services up was facilitated by a ‘spend now, think later’ approach.

*So, we forgot about money. I think all the Trusts in the whole country did at some point. We were just throwing money at a problem because there was nothing else we could do. We forgot about due process because we had to, we had to react fast and now we’re in a position where we have to now explain our financial spend and be able to quantify why we still need this service*. **CS2 Manager**

Existing staff were mobilised on a temporary basis and played a key role in getting services up and running. As many other clinical services were not running as usual, some staff groups were unable to work in their usual role; this enabled existing staff to be mobilised and combined in a way that would not usually be the case when setting up a service. Staff included medical students, clinical academics who had their academic time released by their universities, redeployed, shielding (clinical and administration), and pregnant clinical staff. These kinds of organisational activity at the meso-level represent moments of structural resilience.

### The influence of funding on the delivery and stability of follow-up services

As the scale and pace of the pandemic evolved over the course of 2020 and 2021, local arrangements for enacting situated and structural adaptations were not adequate to address the disruption that was emerging. In England and Wales at governmental level there was oversight and coordination of healthcare in response to the on-going healthcare needs of patients following COVID-19. At the macro-level, systemic adaptations were made to organise system level processes such as funding for services and research, Long Covid leadership and expertise at a national level, national guidelines for management of Long Covid, and service specifications for Long Covid clinics [24]. At the macro level, reorganising sociotechnical resources in this way was necessary for the structures and processes through which sociotechnical resources were organised and delivered at the meso and micro levels. However, how the systemic adaptations were implemented at regional and local level differed between sites.

At CS1 and CS2 systemic adaptations to funding from NHS England resulted in Long Covid monies being allocated to commissioners in the locality, consequently the existing post-hospitalisation service at CS1 was commissioned. Commissioning enabled service planning on a longer-term basis and involved planning, agreeing and monitoring services between clinical service leads in secondary care, a newly appointed Long Covid lead in primary care, and a commissioning lead from the CCG. The commissioning lead took the view that there needed to be a service available to all patients, whether admitted to hospital or not. This required structural adaptations in the form of the redesign and restructure of the initial service delivery model. Commissioning enabled the service to receive referrals for patients whose acute illness had been managed in the community, thereby significantly influencing the situated practices required to deliver the service at the operational frontline. This action was intended to address the significant disruption COVID-19 had on the healthcare needs of patients in the community. However, for the model to be successful, General Practitioners at the operational frontline in primary care needed to be enrolled into the operational activity of the service.

The commissioned nature of the service at CS1 facilitated collaborative working between secondary and primary care, which resulted in the transfer of knowledge from secondary care to primary care through webinars and the development of sociotechnical tools to be embedded in the electronic referral system - an electronic ‘pop-up’ prompt and three-tiered referral system.

*So, without the CCG paying me as a clinical lead [primary care], there isn’t any way that I would have been able to get this work done. If that’s [the service] something that reflects onto other systems, it really does need clinical leadership, you need somebody like [service lead (doctor)] and you need somebody in Primary Care that can oversee the CCG pathway as well and bring in those different elements like digital, how referrals might work and how to motivate Primary Care, doing education sessions for Primary Care, it all starts to fit together once you’ve got really good clinical leadership across both boards, across Secondary and Primary Care*. **CS1 Doctor**

At CS2, the CCG took the decision not to commission the service, meaning it remained a respiratory post-hospitalisation follow-up pathway funded from existing budgets and limited longer-term service planning. At CS3 in the summer of 2021 funding from the Welsh government was provided on a yearly basis specifically for local Health Boards to set up Long Covid services for post-hospitalised and community patients. The decisions made nationally in Wales and at the meso level on how to allocate funding from NHS England meant that funding was protected and recognised as being for the purpose of setting up new services. Structural adaptations permitted by the nature of new investment in services specifically for patients with Long Covid was important for the stability (and situated resilience) of the services at CS1 and CS3. In contrast, at CS2, there was an ongoing requirement to explain the financial spend and be able to demonstrate why the service was needed.

*As they were trying to return to business as usual it got particularly stressful for me to try and keep things going, and as I said, trying to get enough support to keep a clinical fellow in post, so that has been quite stressful, constantly having to prove the need for the service*. **CS2 Service Lead**

### The emergent nature of Long Covid knowledge and expertise on service delivery models

The decisions made by commissioners in England and by the Welsh government on how to allocate funding for services influenced the situated practices at each case site in several ways. The multi-factorial nature of on-going symptoms following COVID-19 became evident from experience of seeing patients and emerging findings from research. As such, multi-disciplinary teams (MDTs) were formed at CS1 and CS3. At CS1, one of the service leads drew upon current and past experiences to set up a virtual weekly MDT meeting.

*I was very aware even from the people that you’re seeing on the acute unit that they had lots of different problems, so we also set up an MDT with other experts and again I suppose I was using what I had learned through developing other services of trying to use the approach where you have one clinician leading a consult but a big MDT involved or multidisciplinary team, meaning both in terms of skillset – so nurses, physio, doctor – but also speciality*. **CS1 Service Lead (Doctor)**

At CS3, the team was recruited with the intention of forming a therapy-led MDT approach, as mandated by the government. Triage and assessment were undertaken by a member of the MDT, patients were then allocated to the most suitable member of the team for treatment, such as an occupational therapy intervention. The decision at the governmental level that the Long Covid service would be therapy-led [23] influenced situated practices. Not all therapists in the new service were competent in holistic patient assessment. As such, the decision at the macro-level created challenges at the micro-level. It took time for therapists new to holistic assessment to learn the skill and as they were learning their assessments took longer to complete. However, moments of situated resilience were created by colleagues with more experience providing training and support.

*So, in our initial assessments, first of all we ask about things like breathlessness and I have no background, and palpitations and heart rate, looking at things like desaturation on exercise, post exertional malaise, we’re looking at their six minute, sit to stand and all of that. I had no idea what any of that meant when I started, that’s a completely different background to me, so it was just about trying to learn enough that if you’re reading something you’re not missing something that’s a red flag*. **CS3 MDT member**

At CS3, in addition to the system level decision made by the Welsh government that the service would be therapy-led, funding to each Health Board was for one service across a large geographical area. Managers decided that structural adaptations would be made to deliver an entirely virtual service. Consequently, in addition to the challenge of learning new skills of holistic patient assessment, this had to be achieved in a virtual setting. From the perspective of staff at the operational frontline, limitations of the virtual service were identified as relationship building between the new team, who had not met in person, and some members of the team reported that the virtual patient assessment and intervention were more suited to some professionals in the team than others.

*It’s hard because over email and when you’re in MDT what you worry is that some of what you’re saying is lost, because they don’t really know you as a person, we all have a really good professional relationship, but none of us really have that additional personal relationship you get working in a team, you know we’re never having lunch together, we’re never really finding out more about each other’s lives*. **CS3 MDT member**

However, staff did acknowledge that the virtual service was potentially an equitable, efficient and cost-effective way to deliver the service by removing the travel requirement for staff and patients.

At CS1 and CS3, the need to view patients holistically and bring in multiple experts enabled clinicians to work cohesively to formulate the most effective plan for patients. The action taken to organise the MDT created moments of situated resilience where staff used their expertise from a range of professions and/or specialisms to advise on patients in the day-to-day running of the service. At CS1, the reach of this action extended to the level of the organisation by creating a more efficient approach to referral between specialities.

*You could see they were going to end up going to a neurologist, a diabetologist, psychologist, you know, multiple specialities which all takes months in the healthcare system and is very expensive, it’s inefficient both for the patient and for the healthcare service. So it’s really obvious in some ways to, when you’re setting these services up, that you just need somebody that’s the lead, somebody that’s saying this patient is mine and I’m going to get to the bottom of everything and then I will reach out and get the help I need to help that*. **CS1 Service Lead (Doctor)**

Collaborative working and joint discussion were viewed as supportive for staff and as improving the quality of the care for patients.

*Long Covid is new and it’s subtle and it waxes and wanes and we’re learning more about it all the time. I think because of those uncertainties about it, the patient can be reassured that they’ve not just been looked at from one prism, one angle of that, they’ve had a multi-aspect perspective and the person who’s leading their care has been challenged or has challenged others about why this couldn’t be X, Y or Z. Also that you’ve been safety-netted, that the person who’s looking after your care knows what to do if things go this way or that, if things change. The GP could be reassured as well, couldn’t they that they don’t now have to refer to psychiatry because we’ve already had that discussion*. **CS1 Member of MDT**

The meetings also served other purposes, including peer support and learning from each other by solving problems through academic and practical discussion, teaching sessions, and the development of decision support tools. Staff also reported that the new knowledge gained was taken back to clinical practice within their specialty.

At CS2 in the early stages of the service, when knowledge of the on-going issues was developing, there was a weekly MDT meeting with radiology. The action of enrolling sociotechnical resources (knowledge, skills, IT) created moments of situated resilience at the micro-level as new knowledge emerged over time.

*We were having a weekly MDT at this point with radiology because we didn’t – not that we didn’t know, we were establishing more firm ways of running that pathway and obviously now in reflection to then, the process is more transparent because we’ve had so many patients come through that have had similar situations so we know kind of how to judge the radiology and the symptoms and what the more long-term effects are of concern*. **CS2 Doctor**

However, as a result of decisions made by commissioners at the meso-level on how to allocate funding from NHS England, CS2 only had funding for a clinical fellow and administrator. This influenced situated practices with specialist review requiring onwards referral to specialities as required.

### Influence of the political and organisational drive to return to business as usual

As the broader context of the pandemic evolved in late 2021 and early 2022, the closure and reduction of services was causing disruption across the health system. In response at the macro level there was a political drive for integration of COVID-19 services with existing services. This meant that services that had been closed or reduced were opened again. At CS1 and CS2 redeployed staff who had been supporting delivery of the services returned to their usual roles, resulting in disruption to the service delivery model. At CS1, because of the service being commissioned, funding was available for replacement staff to be recruited, including administrative staff who played a key role in organising the service.

*I’ve just managed to employ my service coordinator, admin is the key to make sure that these patients are where they need to be on their journey and given the right advice in regards to appointments and things like that, because before that we were utilising secretaries that were already incredibly busy*. **CS1 Service Lead (Nurse)**

At CS2, local leads were cognisant of how vulnerable the lack of commissioned status had left them. Managers who had been involved in discussions with commissioners expressed their frustration at the lack of funding from the CCG for the service and how they had to justify the money being spent.

*The budget closed down in March of 2021 and then all of a sudden people are thinking oh my God, I’m still using all of these staff, I still need all of these pathways in place but it’s costing a huge amount of money. But there was then pressure on our own internal budget codes. From the beginning, in my opinion, it should have been absolutely funded at [commissioning organisation], and then we would have been able to have our model but have a permanently funded one….. As it is, we’re trying to get this extended for a further year*. **CS2 Manager**

*I think it needs to be commissioned as a separate service as opposed to a tag onto respiratory services, because actually again, depending on the time of year and what the variant is, the need will vary and it can’t just be let’s add on several hundred patients to the respiratory clinic. So it needs to be commissioned as a separate pathway, with the right support, with the right admin, with the right access to rehabilitation, psychological support etcetera*. **CS2 Service Lead**

Across all sites, the degree of disruption to the service fluctuated with the impact the different waves and variants had on the number and acuity of people hospitalised with COVID-19. The unpredictable nature of the pandemic created challenges with demand management. Providing a service that was flexible to the changing needs of the pandemic was a challenge.

*We’ve never had a month where we haven’t had 50 discharges, but January 2022 we had 800. So that’s really difficult to actually map and get the service right, so we have had times where there have been quite a lot of delays before people have got to that face-to-face appointment, just because we’ve not been able to match and actually our service, it just isn’t that flexible to allow more clinics at one point, less clinics at another, it’s just not how we’re set up*. **CS1 Service Lead (Doctor)**

At some point for each case study site existing sociotechnical resources were not sustainable to continue responding to the degree of variation in patient numbers, availability of staff and space. This would cause a disruption to on-going activities so adaptations were required in order to create moments of situated resilience at the micro-level. CS2 was located in an area that saw large numbers of patients hospitalised with COVID-19, when redeployed staff returned to their usual roles, because the service was funded from existing budgets, managers and clinicians had to adapt processes in order to manage the demand. Restructuring of resources at CS2 occurred when indicators of potential risk (too many patients for capacity of the service) were triggered. Managers had been monitoring operational activity and the results of QI work were used to inform adaptations to the stratification of patients eligible for the service. The reduction in patients created moments of situated resilience at the micro-level but in turn this had an impact on the reach of the service in terms of patients who would be eligible to access follow-up when they had been hospitalised with COVID-19.

*In January 2021 alone we had 1,389 referrals to the service. In February of the same year, 2021, we decided to change our process because we clearly couldn’t cope with that volume. And this was after the work [QI] being done from perhaps nine months before, so from the May 2020. So there was enough evidence to allow us to change our process, it was clinically led. And what we did is we now only contact people with abnormal radiology. And because of that our figures dropped dramatically. So 1,300 in January 2021, 300 in February 2021, so that makes it more manageable*. **CS2 Manager**

CS3 modelled the service on national and local data on the incidence of COVID-19 and Long Covid. Setting the service up later in the pandemic allowed CS3 to take a pre-emptive and proactive approach with the aim of sustaining the service in the unpredictable context of the pandemic, thereby creating moments of structural resilience.

*I think we had time to understand what we needed to do, although it was a relatively short time to set it up, I think we had that bit of breathing space that probably England didn’t and [what] our Health Board had, is the [time] to step back and not to rush it [setting up], to really, kind of, steps of QI, understand your problem, look at the incidence and take some learning on-board [from] what other people were doing*. **CS3 Service Transformation Lead** (Manager 2).

## Discussion

This study explored the experiences of healthcare professionals and managers of developing and implementing post-hospitalisation follow-up services for people following a hospital admission with COVID-19. In this paper, we have analysed these responses drawing on Macrae’s Moments of Resilience framework.

We found that at each case study site, the needs of patients following a hospital admission with COVID-19 created conditions that required strategies to build resilience into healthcare systems [24, 33]. In response to witnessing the impact of COVID-19 in the acute phase, and demand in the community for specific services for patients experiencing on-going issues, at each case study resource management was required as staff took action to redesign and restructure sociotechnical resources available to them to establish follow-up provision for patients [32]. Actions taken represented the organisation of sociotechnical resources that came together or were used in some way to address a problem, support learning, or to develop some new adaptation or approach to delivering follow-up services [34]. As such, we found similarities and differences in the responses across our case studies.

The response at each case study was influenced by its context and geographical location, and evolved according to the nature of funding, political and organisational factors, particularly when there was a drive to return to business as usual. This response was at all levels of the healthcare system, ranging from clinicians working at the operational frontline, to managers, commissioners and health board leads who oversaw and facilitated the development of new services, and to governmental level where decisions were made about funding and guidance for follow-up services. In this paper, informed by the Moments of Resilience framework of situated, structural and systemic resilience, we have examined the complex interconnections between different levels and scales of complex sociotechnical systems [6].

Our findings show that in the early stages of the pandemic, the scale of change that unfolded around the disruption was at the level of the healthcare organisation [6]. At the micro-level at each case study, before the respective governments had co-ordinated a national level response, the emotional disruption experienced by clinical staff witnessing the on-going issues for patients provoked situated resilience, as staff questioned the safety of current organisational activity - patients being discharged from hospital without follow-up. Clinicians took action to respond to the situated disruption that unfolded by organising sociotechnical resources available to them to detect patients requiring support and adjust working practices to start the process of setting-up some degree of follow-up provision. As events unfolded, situated resilience emerged as clinicians interacted with and assembled sociotechnical resources (knowledge, skills, staff, IT) to identify what needed to be done to set up follow-up provision for patients [35]. For services to be set-up, it was dependant on scaling-up the response from the micro-level to the meso-level. At each case study, the disruption at the micro-level was escalated to the meso-level and enacted resilience at a greater scale of activity - managers were engaged and provided the sociotechnical resources (staff, funding, space, IT) required to set up the service. This exemplifies a general observation in our study, that the usual bureaucratic processes were temporarily eased to enable changes and illustrates the interrelations between different levels in an organisation [6].

Action taken at the meso-level in each case study to facilitate follow-up of patients differed in terms of which existing resources were mobilised, how they were organised, and which patients could be seen in the service. Structural resilience to support work for that particular organisation was created and was a response of adaptation to existing services and resources, in a specific and unique situation that required mediation between the needs of the new follow-up service and the broader organisational constraints created by the pandemic [36]. At CS1, managers were able to provide sociotechnical resources for a comprehensive service, at CS2 the service, beyond the clinical fellow and administrator, was dependant on redeployed staff, and at CS3 managers did not initially consider a service was warranted. Managerial strategies influenced the organisations’ ability to adapt and reorganise system functioning to set up services [37]. However, in the absence of provision for a service, the respiratory consultant at CS3 adapted existing resources to provide a follow-up service that was on a much smaller scale than CS1 and CS2, but did represent a strategy of situated resilience [6]. These insights show that although there were differences, resilience at each case study site was an emergent property of complex adaptive systems and was used to adapt and transform the services in response to the disruption caused by patients requiring follow-up after COVID-19 [38].

At each case study the new service was set up in unprecedented conditions. Leaders recognised the uncertainty and complexity of setting up a new service where the trajectory of on-going issues following COVID-19 was unknown [24]. A strategy to manage this uncertainty and support healthcare delivery was to forge connections and networks among colleagues in their organisations and beyond [6]. Leaders reached out to different professions and specialities with knowledge and expertise beyond their own. Our findings show how collaboration across professional and organisational boundaries created learning processes and resilience [36]. This demonstrates that whilst clinicians were reacting to the disruption they were proactively seeking strategies to create a resilient service that reflected the complexity of the emerging sequelae post COVID-19 [35].

As the pandemic evolved, the disruption to health services from the number of patients with on-going needs was recognised as not being met by existing system-wide arrangements and, if action was not taken, that this would result in an impact on the functioning of the system [39]. As a result, systemic resilience was enacted through the English and Welsh governments creating expert and advisory committees to co-ordinate funding and guidance for clinicians, commissioners and health board leads [6, 24]. Activity unfolded over months rather than years or decades in order to address the rapidly changing context of the pandemic and the emergent healthcare needs of patients with on-going issues. The scale of systemic resilience reached to the meso-level as managers, commissioners and health board leads made decisions about how to allocate funding in their locality to meet the needs of their patient population. These findings show that, as with the emergence of systemic resilience, structural resilience unfolded in weeks and months rather than years.

Over time, as new research emerged and staff gained experience with the clinical and operational requirements of providing a service, multiple layers of processes were enacted which created situated and structural resilience [6]. For example, as a result of limited funding at CS2, large numbers of patients, and a small and unstable team of staff, the fluctuations of the pandemic created a situation where the follow-up service could not meet the demand so structural resilience was enacted and the patients eligible for the service was adapted. At CS1 and CS2 building a resilient system was predominantly reactive action after the disruptive event as a result of the peaks and troughs of the pandemic. The same materially disruptive event occurred at CS3 but to a different extent, so at the meso-level building of a resilient system was predominantly proactive action, before the event [6]. Our findings show that the processes differed between case studies but contributed towards building an infrastructure into each service that was designed to respond to materially disruptive events that occurred as a result of the broader context of the pandemic and its influence on the organisation. This demonstrates how locally situated activities of adjustment and recovery triggered structural change [6].

### Strengths and limitations

This study was aligned to the large PHOSP-COVID platform study, and access to case studies was greatly facilitated by this connection. A potential limitation is that we were therefore restricted to the healthcare organisations taking part within that study. While 64 organisations are represented in PHOSP-COVID, we acknowledge that there are some patterns in these, for example, there is a high number of teaching hospitals. Interviews at CS1 were conducted in autumn 2021, but not at CS2 or CS3 until Spring/Summer of 2022. While we did complete a small number of repeat interviews at CS1, those at CS2 and CS3 were limited to one point in time and relied on participants’ recall of their service’s development from its inception to the time of interview. Finally, we did not interview any participants at the macro-level and therefore our access to this perspective is limited to publicly available documents.

## Conclusion

This paper addresses understudied, yet inherently important, aspects of resilience in healthcare by exploring when and where resilience occurs across the healthcare system and how action taken at one system level influences another. Comparison across the case studies showed that organisations responded in similar and different ways and on varying timescales to a crisis and how national level strategies influenced adaptive capacity across different system levels. Our findings illustrate what strengthened resilient performance in the real world setting of healthcare. As Long Covid is a worldwide challenge for healthcare provision which created significant short - and long-term consequences, further studies could focus on investigating how resilience has been enacted in services based in community as well as secondary care in different locations.

## Data Availability

Data are available from the authors upon reasonable request and following application to the PHOSP-COVID study management board.
